# Hypoxia and Chromatin: A Focus on Transcriptional Repression Mechanisms

**DOI:** 10.3390/biomedicines6020047

**Published:** 2018-04-22

**Authors:** Michael Batie, Luis del Peso, Sonia Rocha

**Affiliations:** 1 Department of Biochemistry, Institute of Integrative Biology, University of Liverpool, Crown Street, Liverpool L697ZB, UK; m.batie@liverpool.ac.uk; 2 Department of Biochemistry, Institute of Biomedical Research, Autonomous Madrid University, Arturo Duperier, 4. 28029 Madrid, Spain; luis.peso@uam.es

**Keywords:** hypoxia, chromatin, transcriptional repression, repressor Complexes, JmjC, histone methylation, HIF

## Abstract

Hypoxia or reduced oxygen availability has been studied extensively for its ability to activate specific genes. Hypoxia-induced gene expression is mediated by the HIF transcription factors, but not exclusively so. Despite the extensive knowledge about how hypoxia activates genes, much less is known about how hypoxia promotes gene repression. In this review, we discuss the potential mechanisms underlying hypoxia-induced transcriptional repression responses. We highlight HIF-dependent and independent mechanisms as well as the potential roles of dioxygenases with functions at the nucleosome and DNA level. Lastly, we discuss recent evidence regarding the involvement of transcriptional repressor complexes in hypoxia.

## 1. Introduction

### 1.1. Hypoxia

Decreases in oxygen availability are generally called hypoxia. These can occur at the organism level such as when climbing high mountains or at the cellular level when oxygen supply is reduced and/or metabolic activity is high [1,2,3]. Changes in response to hypoxia are paramount for cellular and organismic survival [4].

To achieve a cellular response to hypoxia, cells have evolved mechanisms that impinge at all levels of gene expression regulation [2,5] and energy conservation processes. These involve blocks in translation and the cell cycle and switches in metabolic processes such as moving from oxidative phosphorylation to glycolysis [2]. A major coordinator of the cellular response to hypoxia is the Hypoxia Inducible Factor (HIF) transcription factor family.

The HIF family is composed of three different heterodimers including HIF-1β (gene name Aryl Hydrocarbon Receptor Nuclear Translocator (ARNT), shared by all dimers), and HIF-1α, HIF-2α (gene name Endothelial PAS Domain Protein 1 (EPAS1)), and HIF-3α. Oxygen sensitivity is conveyed to HIF via the action of dioxygenases specifically Prolyl Hydroxylase Domain-Containing Proteins (PHDs) and Factor Inhibiting HIF (FIH) [4]. Proline hydroxylation of HIF-α in their oxygen degradation domain creates a high affinity binding site for the tumor suppressor protein von Hippel Lindau (VHL), which is part of the E3-ubiquitin ligase complex containing cullin-2, elongin B/C, and Ring-Box 1 (RBX1) [6]. VHL-dependent ubiquitination signals HIF-α for proteasomal and autophagy mediated degradation [6,7]. However, FIH-dependent hydroxylation of HIF-α, results in an impairment of the recruitment of the key coactivator protein CBP/p300, which is required for a minimum of 40% of all HIF-dependent genes to be expressed [8,9,10,11].

HIF mediated gene expression is largely achieved by direct binding of HIFs to the Hypoxia Response Elements (HREs) present in the regulatory region of target genes [12,13,14,15,16,17,18,19]. However, HIF binding is limited to just a few hundred of the 3.1 million RCGTG motifs present in the human genome [20]. Therefore, chromatin accessibility is one of the major determinants of HIF binding, but not the only one. This provides a potential explanation for the differential genome-wide HIF binding profile and gene expression patterns in response to hypoxia observed across cell lines.

As mentioned above, HIF’s role in coordinating the cell’s response to hypoxia is achieved by transcriptional regulation. For the majority of cases analyzed, HIF is an activator of transcription with very few cases of direct transcriptional repression being described (see below). To activate gene expression, HIF has to engage with chromatin in order to access its DNA binding sites across the genome. As such, chromatin should be considered an important player in the cellular response to hypoxia.

### 1.2. Chromatin

In mammalian cells, DNA is stored as chromatin. Chromatin is a complex and highly dynamic structure containing a mix of DNA and proteins. Chromatin is, therefore, a fundamental regulator of cellular processes requiring access to DNA including DNA-repair, DNA replication, and transcription. As mentioned before, gene transcriptional changes are a key part of the hypoxia response and delineating the crosstalk between chromatin structure and transcription is essential in understanding the cellular response to low oxygen stress.

The structural repeating unit of chromatin is a nucleosome, which consist of 147 base pairs of DNA wrapped 1.65 times around a histone octamer [21,22]. The histone octamer consists of two copies each of the four core histones (Histone (H) 2A, H2B, H3, and H4), which forms as a result of tetramer dimerization between a H3/H4 and a H2A/H2B tetramer [23]. Nucleosomes are linked by linker DNA and linker histone H1 and condense to higher order chromatin structures, which eventually forms chromosomes [24]. There are two major functional states of mature chromatin, heterochromatin, and euchromatin [25]. Heterochromatin is a highly compact state that constitutes a barrier to DNA binding and is associated with silenced loci. Conversely, euchromatin has a more open conformation and is associated with actively transcribing and poised loci. Furthermore, to these two major conformations, microscopy techniques and biochemical assays have shown that chromatin structure is more complex with additional chromatin compaction states [26,27,28]. In *Drosophila* cells, five distinct chromatin states have been identified through DamID assays followed by Chromatin Immunoprecipitation (ChIP) and microarrays [27]. These five states differ in protein binding, histone modifications, biochemical characteristics, and transcriptional activity. Also, researchers using a live cell quantitative Fluorescence Lifetime Imaging (FLIM)–Fluorescence Resonance Energy Transfer (FRET) based assay for chromatin compaction reported three chromatin states based on spatial characteristics [26]. This study also found altered variations in the relative signals of the three types of chromatin state in response to ATP depletion, Trichostatin A (TSA) treatment, and different stages of the cell cycle, which supports previous work on chromatin compaction dynamics. More recently, the aforementioned technique has been used to measure chromatin compaction in the model organism *C. elegans.* The technique found heterogeneous chromatin compaction on the whole organism level with nanoscale spatial and temporal resolution [28]. These studies among others demonstrate the complexity of chromatin organization in metazoan organisms, which indicates the existence of intricate control mechanisms. 

**Figure 1:**
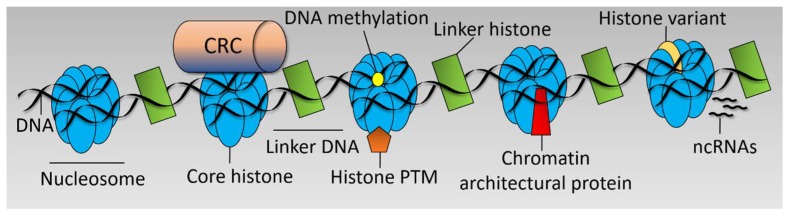
Chromatin structure. Simplified linear diagram of chromatin highlighting the main mechanisms by which chromatin structure is regulated. Chromatin Remodeller Complex (CRC), post translational modification (PTM), and non-coding RNAs, (ncRNAs).

There are various interrelated mechanisms by which chromatin structure is regulated including Chromatin Remodeller Complex (CRC) functions [29], post translational modifications to histones [30], incorporation of histone variants [31], DNA methylation [32], action of non-coding RNAs (ncRNAs) [33], and chromatin architectural proteins [24] (see Figure 1). These mechanisms dictate the chromatin landscape, which is a key determinant in the transcriptional output of the cell and cell fate decisions. Chromatin is responsive to numerous stimuli and developmental cues [34] and is often deregulated in disease [35].

An emerging field is the study of chromatin structure in response to hypoxia where some experimental evidence is now being published.

## 2. Hypoxia-Induced Chromatin Changes

Hypoxia has been shown to induce changes in chromatin structure especially in histone methylation, acetylation, and DNA methylation. In this review, we will focus on methylation.

There is a lack of knowledge pertaining to chromatin compaction states in response to low oxygen stress. Through the use of Single Molecule Localisation Microscopy (SMLM) and in situ DNA digestion coupled with fluorescent microscopy, a rapid change in chromatin architecture and an increase in chromatin compaction has been reported in human cardiomyocytes deprived of oxygen and nutrients [36]. The change in chromatin architecture was found to be rapidly reversible in response to reoxygenation and replenishment of nutrients, which demonstrates the dynamic capacity of chromatin to sense and respond to oxygen and metabolic changes [36]. Another study determined that A431 cancers cells treated with 0.1% oxygen for 48 hours have reduced sensitivity to Mononuclease digestion, which suggests increased heterochromatin composition [37]. Through the use of proteomics, this study also identified an increase in Heterochromatin Protein 1 Binding Protein 3 (HP1BP3) in the chromatin bound fraction of cells treated to hypoxia. HP1BP3 has previously been shown to maintain heterochromatin integrity. Therefore, it could be a player in inducing hypoxic chromatin compaction [37,38].

Chromatin looping, which brings distal sequence regions together, represents additional mechanisms in which transcription is regulated by chromatin architecture [39,40]. The proximal promoter binding at the HRE sites HIF-1α and HIF-2α also bind to intergenic regions of the genome [12,14,41,42] and there is evidence of HIF binding regulating distal gene expression through Promoter Enhancer Interactions (PEIs) [14]. Work from the Ratcliffe and Mole laboratories, utilizing ChIP sequencing and Capture C in MCF7 cells treated to 0.5% for 16 hours, has revealed genome-wide HIF binding-HIF regulated gene PEIs [41]. This study and others also elucidated that HIF promoter binding in hypoxia is predominantly located at pre-established and primed, promoter enhancer loops [41,43]. The results from this study [41] indicate that hypoxia or HIF induction does not alter the chromosome loops identified. However, further analysis is required to establish if hypoxia changes chromosome looping both in a dynamic analysis and in an unbiased manner since the only data available relates to HIF binding sites.

Despite the increase in evidence for chromatin regulation in hypoxia, there is still a great deal of unknowns. The use of imaging and sequencing technologies to study chromatin spatial organization should be used to gain further insight into the dynamic interplay between hypoxia, chromatin, and gene transcription. This would help elucidate how chromatin contributes to gene repression in hypoxia.

## 3. Histone Methylation-Focus on Repression

Histone methylation is a dynamic and reversible post-translational modification at Lysine (K) and Arginine (R) N-terminal tails of histones. These modifications can provide binding sites for chromatin binding proteins and the histone methylation landscape is predictive of the gene transcriptional state, transcription factor binding, and chromatin compaction [44,45]. H3K4, H3K9, H3K27, and H3K36 are among the most common and well-studied histone methylation sites. H3K9 di-methylation (me2)/tri-methylation (me3) and H3K27me3 are linked to transcriptional repression and are key players in cell fate decisions and tissue specific transcriptional control [46]. H3K9me2/3 are markers of heterochromatin and are found at coding and non-coding regions [47]. Both modifications are associated with gene silencing via crosstalk with DNA Methyl Transferases (DNMTs) and recruitment of other chromatin modifying proteins such as Heterochromatin Protein 1 (HP1), which can regulate heterochromatin formation [48]. H3K27me3 is located primarily at gene promoters of open chromatin and is involved in gene repression by recruiting Polycomb Repressive Complexes (PRCs) (reviewed in [49]). H3K27me3 also marks poised enhancers and co-occupies promoters with the active histone modification H3K4me3, these co-occupied sites are termed bivalent [50].

Histone Methyl Transferases (HMTs) add methyl groups to histones, which transfers a methyl group from S Adenosyl Methionine (SAM) (reviewed in [46]). Two families of enzymes remove histone methylations. Lysine Specific Demethylases (LSDs) target H3K4me1/2 and H3K9me1/2 through a Flavin Adenine Dinucleotide (FAD) dependent amine oxygenase reaction [51]. Jumonji-C (JmjC) histone demethylases target a much broader range of histone targets (reviewed in [52,53]). The latter are molecular dioxygenases, which require oxygen, iron, and 2-oxoglutarate for demethylation. This oxygen dependency of JmjC histone demethylases provides an important link to chromatin structure and oxygen sensing [52,53]. The writers and erasers of H3K9 and H3K27 methylation are shown in Table 1.

Hypoxia-induced increases in both active and repressive histone methylations have been shown in several human cancer lines, mouse embryonic fibroblasts, and human tumor samples [54,55,56,57,58,59]. With regard to repressive modifications, H3K9me2 and H3K27me3 increased in Hepa 1–6 cells exposed to 0.2% oxygen for 48 hours [54]. H3K9me3 increases were also observed in RKO cells exposed to 2% and 0.1% oxygen. These changes were rescued upon reoxygenation [59]. Additionally, H3K9me2/me3 increases were shown in mouse macrophages exposed to 1% oxygen for 24 hours [56]. Total H3K9me2/me3 levels were also elevated in A549 cells exposed to 0.5% oxygen as well as site specific increases at the hypoxia repressed gene promoters known as *MutL Homolog 1 (MLH1)* and *Dihydrofolate Reductase 2 (DHFR2)* [58]. Researchers detected increased H3K9me3 after 90 min of 0.5% oxygen exposure. This is the only study which has investigated histone methylation levels in response to acute hypoxia. While the aforementioned studies investigated total levels of histone modifications and some site-specific changes, Prickaerts et al. elucidated specific increases in H3K27me3 and H3K4me3 in response to hypoxia on a genome-wide scale through the use of ChIP sequencing integrated with microarray analysis in MCF7 cells exposed to oxygen deprivation and reoxygenation [57]. Many of these changes were reversible upon reoxygenation and showed correlation with transcriptional changes in hypoxia. The researchers uncovered evidence for hypoxia acquired promoter bivalency, which modulated poised/active gene transcriptional control. Xenografts of human breast and lung cancer were also found to have increased H3K27me3 and H3K9me3, respectively [57,59].

Mechanistically, hypoxic induction of histone methylation levels has been attributed to inhibition of JmjC histone demethylases [57,58]. JmjC histone demethylases are oxygen-dependent enzymes and also have a catalytic fold similar to that of FIH [60,61]. While oxygen dependency of the majority of JmjC enzymes has not been established, in vitro histone peptide methylation assays and histone methylation assays from cell lysates have demonstrated the potential of some of these enzymes to function as bona fide oxygen sensors [57,58,62,63]. Among these, the H3K27 demethylase KDM6B and the H3K9 demethylase KDM4E were inhibited over physiologically relevant oxygen concentrations with the latter displaying similar oxygen dependency kinetics to PHD2 [63]. The H3K9 demethylase KDM4A has a reported km for oxygen of 173 ± 23 µM, which places it between FIH and PHDs with regard to oxygen dependency [62]. Furthermore, increased H3K9me3 in U2OS cells overexpressing KDM4A was reduced in a stepwise fashion when exposed to 5%, 1%, and 0.1% oxygen [62]. These studies demonstrate how JmjC histone demethylases can function as cellular oxygen sensors.

Many JmjC histone demethylases are transcriptionally upregulated in hypoxia with some as direct HIF target genes [53,64]. It is speculated that upregulation of JmjC histone demethylases in response to low oxygen may be a feedback mechanism to help maintain histone methylation status of the cell. However, it should be noted that there is evidence for JmjC histone demethylases remaining active in response to low oxygen stress, with their demethylation activity mediating hypoxia induced transcriptional changes. [65,66]. KDM4C and KDM3A were found to bind HIF-1α and function as enhancers of HIF-1α transactivation activity in hypoxia via H3K9 demethyation at hypoxia responsive promoters [65,66]. Therefore, determining the dynamics of JmjC histone demethylase oxygen sensitivities as well as activities in different oxygen environments and cell backgrounds is needed. Non-histone targets and histone methylation independent functions of JmjC histone demethylases should also be considered when investigating their possible roles in hypoxia-induced transcriptional repression [53].

Pertaining to HMTs and histone methylation in hypoxia, G9α (gene name Euchromatic Histone Lysine Methyltransferase 2 (EHMT2)) protein levels are induced post transcriptionally in hypoxia [58]. G9α was recently identified as a PHD1 target for hydroxylation and is degraded in a VHL dependent manner [67]. Moreover, via promoter H3K9me2, G9α mediates transcriptional repression at a subset of hypoxia-repressed genes and there is growing evidence for chemotherapeutic benefit in targeting G9α through dysregulation of hypoxic gene expression [67,68,69]. These studies indicate that G9a inhibition reduces oncogenic responses such as angiogenesis, proliferation, and survival [67,68,69].

SET Domain Bifurcated 1 (SETDB1) and Suppressor of Variegation 3–9 Homolog (SUV39H) 2 protein levels are also stabilized and are low oxygen stress and hypoxia-induced. SETDB1 mediated H3K9me3 upregulation on the Ataxia Telangiectasia Mutated (ATM) and p53-Associated KZNF Protein (APAK) gene reduces its expression [70]. This triggers an increase in p53-dependent hypoxia-induced apoptosis. Manipulation of this pathway deregulates cell viability in hypoxia [70]. Elevated H3K9me3 in hypoxia is also required for ATM activation in the absence of DNA damage, which facilitates DNA replication in a low-oxygen environment [59]. This study suggests a mechanism for H3K9me3-mediated activation of ATM in hypoxia involving transcriptional repression of ATM-specific phosphatases including Protein Phosphatase 2 (PP2A). Developmental importance of H3K9 methylation and hypoxia is shown by SUV39H1 and SUV39H2, which are hypoxia-inducible. Loss of their expression is associated with the epigenetic changes required during fetal lung development [71].

Given the recent evidence of hypoxia-induced histone methylation changes, further analysis on how histone methylation contributes to the transcriptional repression observed in hypoxia is therefore necessary.

## 4. Chromatin Remodelers in Hypoxia-Focus on Repression

**Figure 2:**
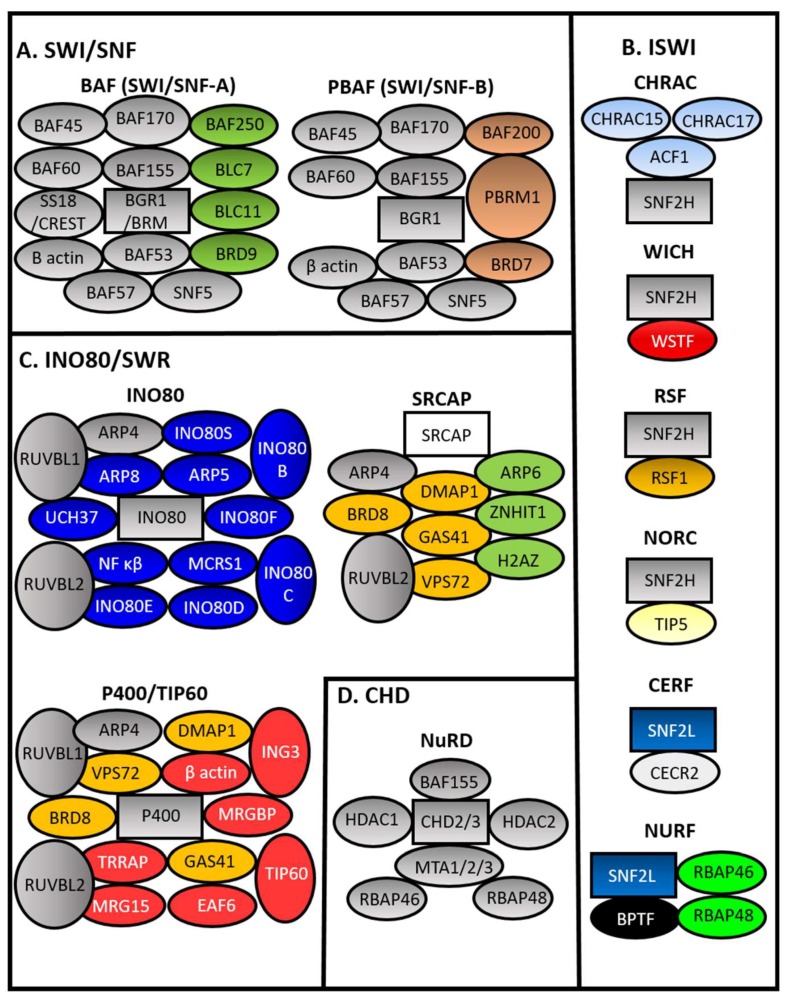
Mammalian Chromatin remodeler complexes. Chromatin Remodeler Complexes (CRCs) from the four subfamilies of CRCs based on ATPase domains are shown. (**A**) SWItch/Sucrose Non-Fermentable (SWI/SNF), (**B**) imitation SWI (ISWI), (**C**) inositol-Requiring 80/Sick with RSC (INO80/SWR), and (**D**) Chromodomain Helicase DNA-binding CHD (CHD). Rectangles represent ATPase domain. Brahma (BRM) BRM Related Gene 1 (BRG1), BRG1/BRM Associated Factor (BAF), Polybromo Associated BAF Complex (PBAF), Snf2 Related CREBBP Activator Protein (SRCAP), Tat Interacting Protein 60 (TIP60), Nucleosome Remodeling Deacetylase (NuRD), Chromatin Accessibility Complex (CHRAC), Remodeling And Spacing Factor (RSF), Nucleolar Remodeling Complex, Cat Eye Syndrome Chromosome Region Candidate 2 (CERC2), CECR2 Containing Remodeling Factor (CERF), Nucleosome Remodeling Factor (NURF), B-Cell CLL/Lymphoma (BCL), Bromodomain Containing (BRD), Polybromo 1 (PBRM1), RuvB Like AAA ATPase (RUVBL)(RVB), Actin related protein (ARP), Ubiquitin C-Terminal Hydrolase L5 (UCH37 Microspherule Protein 1 (MCRS1), Nuclear Factor Kappa B Subunit (NF κβ), DNA Methyltransferase 1 Associated Protein 1 (DMAP1), Glioma-Amplified Sequence 41 (GAS41), Vacuolar Protein Sorting 72 (VPS72), Zinc Finger HIT-Type Containing 1 (ZNHIT1), H2A Histone Family Member Z (H2AZ), Transformation/Transcription Domain Associated Protein (TRRAP), MORF-Related Gene 15 Protein (MRG15), MRG Domain Binding Protein (MRGBP), Esa1 Associated Factor 6 (EAF6), Inhibitor Of Growth Family Member 3 (ING3), Histone Deacetylase, (HDAC), Metastasis Associated (MTA), Retinoblastoma-Binding Protein (RBAP), ATP-Dependent Chromatin Assembly Factor 1 (ACF1), Sucrose Nonfermenting Protein 2 Homolog (SNF2H), Sucrose Nonfermenting 2-Like Protein 1 (SNF2L), Williams Syndrome Transcription Factor (WSTF), and Bromodomain PHD Finger Transcription Factor (BPTF).

Hypoxia engagement with chromatin remodelers has been analyzed in the context of transcriptional activation. Four main families of chromatin remodelers can be found in mammalians. These are the SWItch/Sucrose Non-Fermentable (SWI/SNF), Chromodomain Helicase DNA-binding (CHD), Inositol-Requiring 80 (INO80), and Imitation SWI (ISWI) families (see Figure 2) [52,53]. Many more sub-complexes exist, which gives rise to increased complexity in function and regulation [72].

Regarding the hypoxia signaling pathway, the SWI/SNF family seems to be particularly important with a high level of human mutations found in renal clear cell cancer where VHL is also found to be highly mutated [73]. The SWI/SNF complex (see Figure 2A) can be subdivided into two sub-complexes called BRG1/BRM Associated Factor (BAF) and Polybromo Associated BAF Complex (PBAF). These are defined not only by their catalytic subunits (Brahma (BRM) or BRM Related Gene 1 (BRG1)) but also by differences in assessor factors such as BAF250/BAF250B (BAF) and Polybromo 1 (PBRM1) (PBAF) [74].

It is known that hypoxia engages and requires SWI/SNF for activation of HIF and its targets [53,75]. However, there is no indication so far that SWI/SNF can be involved in hypoxia-mediated repression. A few studies have identified repression functions associated with BAF [76,77] and PBAF complexes [76,78,79]. From these studies, one can speculate mechanisms by which hypoxia could engage with SWI/SWF for repressing genes. For example, it was shown that the Repressor Element 1-Silencing Transcription Factor (REST), CoREST transcription factors, and known repressors require SWI/SNF components for repression of genes in neuronal settings although no discrimination between BAF and PBAF was provided in this study [76]. Interestingly, REST has recently been shown to mediate a significant proportion of hypoxia-induced gene repression [80]. An additional study determined that PBAF is required for transcriptional repression in response to DNA damage [79]. In particular, this study demonstrated that PBRM1 was phosphorylated by ATM, which is a modification that was necessary for this repressive activity of PBAF [79]. A connection with hypoxia could be speculated since hypoxia is known to activate ATR/ATM in several cell types [81]. However, as mentioned before, all of these possible scenarios have not been formally studied or demonstrated.

Perhaps the chromatin remodeling family most often associated with transcriptional repression is ISWI (see Figure 2B). In mammalians, it is subdivided into several additional complexes depending on assessor partners for the two catalytic subunits Sucrose Nonfermenting Protein 2 Homolog (SNF2H) and Sucrose Nonfermenting 2-Like Protein 1 (SNF2L) (see Figure 2B). ISWI is known to control nucleosome-spacing and this has been shown to have a cooperative action with the CHD family of remodellers [82]. ISWI also associates with additional factors and is important for the function of CCCTC-Binding Factor (CTCF) in establishing chromatin barriers [83].

ISWI has been shown to contribute to the cellular response in hypoxia by controlling levels of FIH and, therefore, some of the HIF-1 dependent targets [84]. In addition, it also controls the levels of FIH-independent targets, which suggests a broader role in transcriptional regulation in hypoxia [84]. Interestingly, ISWI was identified as part of a complex containing the transcriptional corepressor C-terminal Binding Protein (CtBP) [85]. CtBP has been associated with the transcriptional response to hypoxia through a variety of studies [86,87,88,89,90]. It is, therefore, possible that ISWI action in hypoxia is connected to CtBP. However, this has not been formally addressed.

INO80 family (Figure 2C) is comprised of a large family of remodelers with the catalytic subunits INO80 and Snf2 Related CREBBP Activator Protein (SRCAP), which are characterized by Rec-like helicase domains [91]. This family is known to be involved in nucleosome sliding and histone exchange [92]. Associations with the hypoxic response have been found, but whether the chromatin remodeling aspect of the complex is required is still not clear. Components of the Tip60 complex, more specifically Pontin (gene name RuvB Like AAA ATPase (RUVBL)1) and Reptin (RUVBL2), have been associated with the modulation of the cellular response to hypoxia. In hypoxic conditions, Reptin is methylated at lysine 67 by the methyltransferase G9α and this modification allows it to bind to HIF-1α. Its recruitment to the promoters of hypoxia-responsive genes is where it negatively affects their transcription [93]. Consequently, Reptin deletion results in enhanced induction of a subset of HIF targets, which suggests that the axis G9α /Reptin may work as a negative feed-back loop that acts to limit HIF activity. However, since these studies focused on the role of Reptin on genes upregulated by HIF, its potential role on gene repression under hypoxia remains unexplored. Similarly, methylation of Pontin by G9α induced by hypoxia also potentiates HIF-mediated activation [94]. However, no data was provided regarding the role of Pontin in hypoxia-mediated repression in this study.

At present, there are no studies concerning the role of CHD family in the response to hypoxia.

## 5. DNA Methylation in Hypoxia

One of the most studied aspects of chromatin changes and transcriptional repression mechanisms is DNA methylation. DNA methylation classically occurs at CpG islands present at promoters, which leads to inhibition of promoter activity by failing to recruit specific transcription factors or by active recruitment of transcriptional repressor complexes [95]. DNA methylation is set by DNMTs of which three have been described in humans. These are known as DNMT1, DNMT3A, and DNMT3B [96,97]. As with most processes in the cell, DNA methylation is a reversible state through the involvement of specific enzymes called Ten-Eleven-Translocation (TET) [5,96]. These enzymes remove DNA methylation by hydroxylation of 5-methylcytosine, which is followed by further potential oxidation reactions. These additional modified bases are removed by thymine DNA glycosylase and base excision repair pathways [5].

DNA methylation in hypoxia has been recently studied due to the finding that TET (TET1, TET2, and TET3) enzymes are dioxygenase enzymes, which require oxygen, 2-oxoglutarate, and iron for their catalytic activity [5]. A study using in vitro models as well as tumor hypoxia in patient samples demonstrated that hypoxia does alter global levels of DNA methylation irrespective of proliferation or metabolism [98]. This study showed that hypoxia increased DNA methylation across promoters in a manner that was dependent on TET enzymatic activity [98]. Therefore, TET enzymes require oxygen for their function. By default, hypoxia should increase DNA methylation across the genome.

**Figure 3:**
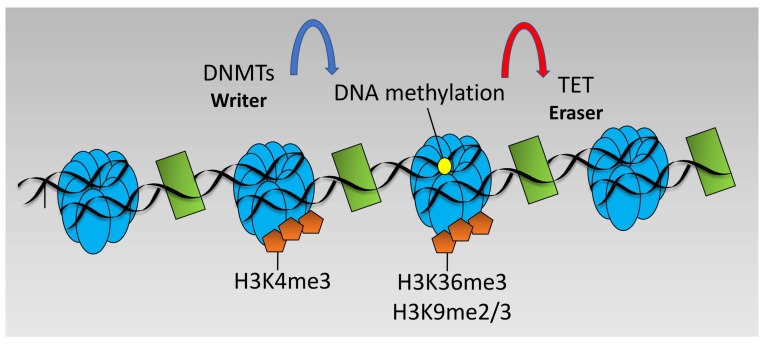
Dynamics of DNA methylation. DNA methylation is performed by DNA Methyl Transferases (DNMT) enzymes and removed by Ten-Eleven-Translocation (TET) enzymes. DNMTs target areas of unmethylated H3K4 and trimethylated H3K36 or di/trimethylated H3K9.

DNA methylation changes at particular loci may also be altered by hypoxia in an indirect manner via altered recruitment of DNMTs. For example, DNMT3 is targeted to loci marked with unmethylated H3K4 or H3K36me3 [96] and H3K9 methylation is strongly linked to DNA methylation via DNMT recruitment [48]. Since hypoxia induces H3K4me2/me3, H3K36me3, and H3K9me3 [53], it is possible to speculate that hypoxia would lead to decreased recruitment of DNMT3 and DNA methylation at promoters marked H3K4me3 but would increase recruitment and DNA methylation across gene bodies marked with H3K36me3 and loci marked with H3K9me2/me3 (see Figure 3). However, such studies have not been performed and further investigation is required for this to be formally demonstrated.

## 6. HIF-Dependent Mechanisms of Repression

As mentioned above, in contrast to gene upregulation, the mechanisms by which hypoxia leads to gene repression are not well understood. In some sporadic cases, direct HIF binding could mediate the transcriptional repression, which was suggested for the regulation of Carbamoyl-Phosphate Synthetase 2, Aspartate Transcarbamylase, And Dihydroorotase (*CAD*) [99], Alpha Fetoprotein (*AFP*) [100], Adenosine Kinase (*AK*) [101], Cystic Fibrosis Transmembrane Conductance Regulator (*CFTR*) [102], and Adenomatous Polyposus Coli (*APC*) [103] genes. It has been proposed that HIF binding could displace transcriptional activators such as the MYC Proto-Oncogene and the BHLH Transcription Factor (MYC) or recruit co-repressors that could account for down-regulation of gene expression [104]. However, these cases appear to be sporadic since genome-wide mapping of HIF-binding sites was unable to detect a significant association between gene repression and proximal HIF binding [12,13,15,16,17,18,19]. Interestingly, knock-down of HIF isoforms prevents the majority of the effects of hypoxia on both gene induction and repression [12,105]. Therefore, for the large majority of genes, hypoxia-triggered gene repression is likely to be indirectly mediated by HIF likely through trans-acting elements. In keeping with this hypothesis, HIF-1α directly regulates the expression of several sequence-specific repressors such as the MAX Interactor 1 and the Dimerization Protein (*MXI1*) gene, which encodes a repressor of *MYC* and leads to the repression of MYC targets such as PPAR-Gamma Coactivator 1-Beta (PGC-1β [106]. Similarly, the transcriptional repressors Basic Helix-Loop-Helix Family Member E40 (*BHLHE40*) [107] and BTB Domain And CNC Homolog 1 (*BACH1*) [108] are direct HIF targets. *BHLHE40* induction by hypoxia results in the repression of Peroxisome Proliferator Activated Receptor Gamma 2 (*PPARG2*) [109], Sterol Regulatory Element Binding Transcription Factor 1 (*SREBP1)* [110], Signal Transducer And Activator Of Transcription 1(*STAT1*) [111], and Melanogenesis Associated Transcription Factor (*MITF*) [112] genes while induction of *BACH1* results in Heme Oxygenase 1 (*HMOX1*) repression [108]. Although these individual gene studies suggest a role for these repressors in the response to hypoxia, their relative contribution to the global transcriptional repression has only been analyzed for MXI1 [113,114]. These studies found that *MXI1* knockdown had very little effect on the hypoxic transcriptome, which suggests functional redundancy with other repressors of the Mad family. Alternatively, it could be that MXI1 is just one of many repressors downstream of HIF with each one acting on a small fraction of the genes repressed by hypoxia. Additional studies are therefore required to fully answer this question.

BHLHE40 was shown to inhibit myogenic differentiation in response to hypoxia through the induction of Myogenin (*MYOG*) independently of HIF [115], which suggests that BHLHE40 has a major role in hypoxia-triggered gene repression acting in pathways both HIF-dependent and independent. In addition to BHLHE40, REST accumulates in the nucleus in response to hypoxia and acts as a key repressor of the hypoxic transcriptome in an HIF-independent manner [80].

Lastly, several studies have established a role of hypoxia and HIF in regulating specific microRNAs particularly miR-210, which act to repress gene expression by inducing mRNA decay and/or inhibiting their translation [16,116,117]. Therefore, at least part of the gene repression observed under hypoxia could occur at the post-transcriptional level.

## 7. Transcriptional Repression Complexes in Hypoxia

### 7.1. SIN3A-HDAC

Although it is known that HIF recruits Histone Deacetylases (HDACs) and HDAC inhibitors prevent HIF-mediated transcription [118], little attention has been paid to the role of these co-repressor complexes in the transcriptional response to hypoxia. The class I HDACs called HDAC1, HDAC2, and HDAC3 are ubiquitously expressed nuclear enzymes that are components of multiprotein repressor complexes. These include SIN3 Transcription Regulator Family Member A (SIN3A), nucleosome-remodeling HDAC (NuRD), and CoREST. The two highly related HDAC3-containing complexes are known as nuclear receptor co-repressor (NCoR or NCOR1) and silencing mediator of retinoic acid and thyroid hormone receptor (SMRT or NCOR2) [119]. However, to date, the only HDAC-containing co-repressor complex whose function in hypoxia has been analyzed at global scale is SIN3A.

The SIN3 protein is highly conserved from yeast to mammals. It is a central component of the SIN3 corepressor complex that participates in a wide variety of processes including development, energy metabolism, cell growth, and differentiation as well as several pathological conditions such as oncogenic transformation [120]. In mammals, there are two SIN3 isoforms known as SIN3A and SIN3B, which are encoded by separate genes, are widely expressed, and bind common as well as distinct transcriptional repressors and complexes [120].

A recent bioinformatics approach found that SIN3A was overrepresented in the proximity of genes whose transcription is repressed by hypoxia [114]. This study also identified enrichment for HDAC1, HDAC2, and Sap30, which are all components of the SIN3A co-repressor complex. The sequence-specific repressors called MXI1, Max, E2F4, and E2F6 are known to interact with the SIN3A complex. In agreement with these computational predictions, knock-down of SIN3A significantly attenuated the repression of over 75% of the genes that were down-regulated in control cells, which suggests a major role for the co-repressor complex in this process [114]. However, several lines of evidence indicate that the function of SIN3A in the control of transcription is more complicated than previously anticipated. On the one hand, the genome-wide binding pattern of SIN3A showed a strong enrichment for this factor in active promoter regions with a SIN3A signal centered at the transcription start sites of actively transcribed genes and absent from genes with low or undetectable expression. On the other hand, SIN3A depletion not only affected gene repression, but it also diminished the induction of about 47% of the genes upregulated by hypoxia. These results suggest that, beyond its function as co-repressor, SIN3A has a wider role in transcriptional regulation. SIN3A was initially described in yeast as a protein with dual functions of being an activator and a repressor [121,122] and recent studies are putting forward its role as an activator of specific genes [92] including targets of the aryl hydrocarbon receptor (AHR), which is a transcription factor related to HIF known to mediate the transcriptional response to xenobiotics [123]. Moreover, the dual role of SIN3A in transcriptional repression and activation in response to hypoxia is not unique to this repressor complex. It has been reported that knock-down of the repressor HEXIM1 affects a similar number of genes repressed and induced by hypoxia [124]. Furthermore, close analysis of results obtained by Casciello et al. [67] indicate G9α also possesses dual functions. In fact, emerging models propose that recruitment of both co-repressor and co-activator complexes is needed for gene induction [125]. As such, further scrutiny is required to understand how these complexes promote or repress transcription at particular target genes.

### 7.2. REST

Perhaps the transcription silencer mostly associated with hypoxia in recent years has been REST. Initially associated with neuronal development [126], its functions have now been extended to multiple cell types including cancer cells [127], cardiac cells [128], and beta cells [129].

REST mediates transcriptional repression via several mechanisms. Via its zinc fingers, it is able to bind to repressor elements. Via its N-terminus, it recruits SIN3A complex and via its C-terminus recruits CoREST, HDACs, LSD1, G9α, and a Methyl-CpG Binding Protein 2MeCP2 [129]. A role for CoREST and its associated histone demethylase LDS1 has been described for the repression of the gene *MLH1*, which is a key component of the DNA mismatch repair system in response to hypoxia [130]. The CoREST complex is recruited by the REST sequence-specific factor to repressed genes and, in addition to LSD1, CoREST complexes also interact with the G9α histone methylase. Therefore, given the role of CoREST in *MLH1* down-regulation and the effects of REST and G9α in the repression of genes under hypoxia described above, it would be interesting to investigate the effect of this co-repressor in the global response to hypoxia.

REST was shown to be repressed by a hypoxia-induced micro-RNA, miR-106 b~25 cluster, in advanced prostate cancer [131]. However, more recent studies have shown that REST is involved in transcriptional repression in hypoxic conditions [80,132,133,134]. Cavadas et al. [80] demonstrated that hypoxia can induce nuclear translocation of REST and REST is required for the repression of around 20% of hypoxia repressed genes in HEK293 cells. This data indicates the importance of this transcriptional repressor but also highlights that other mechanisms are vital for transcriptional repression in hypoxia. Furthermore, it is tempting to speculate that REST’s importance in contributing to transcriptional repression following hypoxia might be dependent on the cell type. Given the importance of REST in neural and cardiac tissue (two tissue very sensitive to changes in oxygen supply), it is possible that REST contribution to the hypoxia response in these tissues could be even more prevalent. However, further studies are necessary to establish if this is the case.

## 8. Conclusions

**Figure 4:**
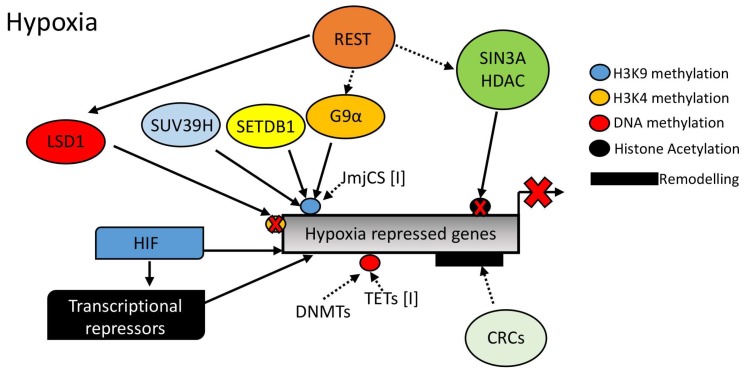
Mechanisms of hypoxic gene repression in the context of chromatin structure. Hypoxia Inducible Factor (HIF), Repressor Element 1-Silencing Transcription Factor (REST), SIN3 Transcription Regulator Family Member A (SIN3A), Histone Deacetylase, (HDAC), G9α Like Protein 1 (GPL), Suppressor of Variegation 3–9 Homolog (SUV39H), SET Domain Bifurcated 1 (SETDB1), Chromatin Remodeller Complex (CRC), Jumoni C (JmjC), DNA Methyl Transferase (DNMT), Ten-Eleven-Translocation (TET), and Lysine Specific Demethylase 1 (LSD1).

As mentioned above, although much is known regarding hypoxia-induced gene expression, far less is known concerning the mechanism underpinning hypoxia-induced gene repression (see Figure 4). With the discovery of a variety of dioxygenases impinging on several aspects of gene regulation such as histone methylation and DNA methylation, it could be hypothesized that these dioxygenases are involved in transcriptional repression in response to reduced oxygen. However, further studies are needed to address this.

Repressor complexes have been associated with the regulation of the hypoxia response. However, these are still just the tip of the iceberg. While H3K27me3 levels are induced by hypoxia [54,57] and H3K27me3 is associated with Polycomb repressive complexes (PRCs) [49], there is no evidence of gene silencing mechanisms being activated via PRCs in response to hypoxia. This lack of information is also extended to chromatin remodelers associated with repression of transcription. As such, much more work is needed to determine the mechanism(s) of hypoxia-mediated gene repression.

## Figures and Tables

**Table 1:** Writers and erasers of H3K9 and H3K27 methylation. Histone Methytransferases (HMTs), Jumonji-C (JmjC) histone demethylases, and Lysine Specific Demethylases (LSDs) targeting H3K9 and H3K27 are shown (targets are in brackets). Euchromatic Histone Lysine Methyltransferase 2 (EHMT2, G9α), G9α Like Protein 1 (GPL), Suppressor Of Variegation 3–9 Homolog (SUV39H), SET Domain Bifurcated 1 (SETDB1), PR/SET Domain (PRDM), Enhancer Of Zeste 2 Polycomb Repressive Complex 2 Subunit (EZH2), Lysine Demethylase (KDM), PHD Finger Protein (PHF), and Myc-Induced Nuclear Antigen (MINA).

Writer	Eraser	
HMTS	JmjC Histone Demethylases	LSDs
G9α (H3K9/H3K9me1/me2)	KDM3A (H3K9me1/me2)	LSD1 (H3K9me1/me2)
GL9 (H3K9/ H3K9me1/me2)	KDM3B (H3K9me1)	(H3K4me1/me2)
SUV39H1 (H3K9me1/me2)	KDM4A (H3K9me2/me3)	LSD2
SUV39H2 (H3K9me1/me2)	KDM4B (H3K9me2/me3)	(H3K4me1/me2)
SETDB1 (H3K9)	KDM4C (H3K9me2/me3)	
PRDM2 (H3K9)	KDM4D (H3K9me2/me3)	
PRDM3 (H3K9)	KDM4E (H3K9me2/me3)	
PRDM6 (H3K9)	KDM6A (H3K27me2/me3)	
EZH2 (H3K27/H3K27me1/me2)	KDM6B (H3K27me2/me3)	
	KDM7A (H3K9me1/me2)	
	(H3K27me1/me2)	
	PHF2 (H3K9me1/me2)	
	(H3K27me1/me2)	
	PHF8 (H3K9me1/me2)	
	MINA (H3K9me3)	
